# An exploration of EEG features during recovery following stroke – implications for BCI-mediated neurorehabilitation therapy

**DOI:** 10.1186/1743-0003-11-9

**Published:** 2014-01-28

**Authors:** Darren J Leamy, Juš Kocijan, Katarina Domijan, Joseph Duffin, Richard AP Roche, Sean Commins, Collins, Tomas E Ward

**Affiliations:** 1 National University of Ireland Maynooth, Maynooth, Co., Kildare, Ireland; 2 Jozef Stefan Institute, Jamova 39, SI-1000 Ljubljana, Slovenia; 3 University of Nova Gorica, Vipavska 13, SI-5000 Nova Gorica, Slovenia; 4 Adelaide and Meath Hospital, Tallaght, Dublin, Ireland

**Keywords:** BCI, Stroke rehabilitation, EEG, CSP

## Abstract

**Background:**

Brain-Computer Interfaces (BCI) can potentially be used to aid in the recovery of lost motor control in a limb following stroke. BCIs are typically used by subjects with no damage to the brain therefore relatively little is known about the technical requirements for the design of a rehabilitative BCI for stroke.

**Methods:**

32-channel electroencephalogram (EEG) was recorded during a finger-tapping task from 10 healthy subjects for one session and 5 stroke patients for two sessions approximately 6 months apart. An off-line BCI design based on Filter Bank Common Spatial Patterns (FBCSP) was implemented to test and compare the efficacy and accuracy of training a rehabilitative BCI with both stroke-affected and healthy data.

**Results:**

Stroke-affected EEG datasets have lower 10-fold cross validation results than healthy EEG datasets. When training a BCI with healthy EEG, average classification accuracy of stroke-affected EEG is lower than the average for healthy EEG. Classification accuracy of the late session stroke EEG is improved by training the BCI on the corresponding early stroke EEG dataset.

**Conclusions:**

This exploratory study illustrates that stroke and the accompanying neuroplastic changes associated with the recovery process can cause significant inter-subject changes in the EEG features suitable for mapping as part of a neurofeedback therapy, even when individuals have scored largely similar with conventional behavioural measures. It appears such measures can mask this individual variability in cortical reorganization. Consequently we believe motor retraining BCI should initially be tailored to individual patients.

## Background

Brain computer interfaces (BCI) have been suggested as a means by which neuro-rehabilitation following stroke may be enhanced [1-6]. EEG-based BCI in particular are the focus of current endeavours. As many stroke patients suffer complete paralysis of a limb, this non-invasive physiological measurement modality provides a means through which brain activity associated with motor control can be monitored, even in the absence of the normal behavioural information provided by the movement itself. It is conjectured that rehabilitation therapy may be effectively administered to patients incapable of movement through the provision of feedback on their attempt to move as determined by the BCI.

Communication and control BCIs based on motor paradigms typically aim to decode EEG patterns to allow a user to learn to control an external device, such as a computer or motorised wheelchair, in the absence of motor control [7-10]. In this study, however, we are interested in overt and attempted movement of a subject, as the ultimate goal of the rehabilitative BCI modality considered here is to train a patient to regain control of the affected appendage. A rehabilitative BCI in this context should encourage and reward the subject for attempted movement, to encourage positive neuroplastic changes in the brain and facilitate recovery of motor control [11]. Such an approach is subtly different from the motor imagery BCI paradigm also applied in stroke rehabilitation. Under the motor imagery paradigm the BCI is used to provide feedback to the patient on their engagement with motor imagery tasks. Motor imagery requires that the patient engage in a mental rehearsal of the targeted movement without attempting to actually execute the movement. It has been suggested that such an approach can supplement conventional therapy for certain patient groups [12]. The work here is predicated on the attempted movement paradigm, which seeks to provide positive reinforcement feedback in response to successful engagement of the patient’s motor networks associated with the targeted motor task. It is speculated that such an approach can help reduce the possibility of the learned non-use phenomenon through the delivery of contingent rewards - a form of neurofeedback therapy [13].

There are significant engineering obstacles to the achievement of such a goal however. These challenges are, in many respects, similar to those encountered by researchers attempting to make BCI more usable for healthy subjects for the purposes of communication and control. Conventional BCI design requires attention to usability issues such as reducing setup complexity by minimising the number of electrodes required, reducing training time and lightening the cognitive workload associated with operation. These aspects must be satisfied while maintaining useful function – not an easy task as it requires maintenance of robust performance in the face of poor instrumentation setup, artefact-inducing subject movement and other detrimental factors.

These problems present an even greater barrier to adoption of this technology when considered in the context of stroke rehabilitation due to the impact of the condition on the abilities of the user. A typical stroke sufferer for whom this technology is potentially useful will obviously have very limited ability to manipulate a device precisely and accurately on to their head unaided and therefore any solution must be tolerant to such setup errors. In addition it is well established that stroke sufferers fatigue very easily [14-17] and therefore in order to maximize therapy during a session minimal (and ideally zero) time should be lost to training the classifier.

Finally, as stroke is an injury to the brain, the stereotypical patterns of brain activity upon which conventional BCI paradigms rely are not guaranteed to manifest themselves conventionally in response to movement intentions and therefore it is not clear how best the BCI should use the signals presented by the user. To compound this latter aspect further, it is not clear how the EEG of the recovering brain will resolve over time, which has ramifications in terms of pattern recognition and subsequent interpretation.

The purpose, of the study reported in this paper, is focussed on this latter aspect. We perform a comparative exploratory analysis of the reliability and stability of motor-related EEG features in stroke subjects from a machine learning perspective. We wish to explore if such features are sufficiently universal that machine learning parameters trained using healthy subjects can be used for stroke-affected patients and further if these remain useful and valid during the critical period of recovery bridging the sub-acute to chronic phases. If BCI trained with healthy stereotypical data provides sufficiently good performance with stroke sufferers then such a deployment paradigm would make BCI for stroke rehabilitation far more practical in a clinical setting. If, on the other hand, the stroke-affected EEG presents sufficiently differently from healthy EEG or that it changes over time, the practical application of BCI in such a context will require more sophisticated design from a machine learning perspective. The study here attempts to shed some light on this pragmatic issue.

## Methods

### Subjects

Fifteen subjects in total participated in the study. Ten subjects were healthy while five were stroke patients. The healthy subjects (8 men and 2 women, mean age 57.2±17.6 years) each participated in one recording session. The stroke subjects (3 men and 2 women, mean age 59.0±9.4 years) participated in two recording sessions. The average time from stroke to the first recording session was 22.2±12.9 days. The average time between first and second session was 190.6±26.1 days.

Stroke patients were recruited from the Adelaide & Meath Hospital, Dublin while control subjects were recruited from the National University of Ireland Maynooth. Inclusion criteria for the stroke patients is detailed elsewhere [18] and summarised here as: Patients must *(1)* be cognitively high functioning, *(2)* be able to give informed consent and follow experimental instructions, *(3)* not suffer from a visual field defect or visual neglect, and *(4)* have upper limb motor paresis in either their dominant or non-dominant hand.

When possible, the Mini Mental State Exam (MMSE) was used to ensure absence of serious cognitive impairment in the stroke patients. One subject was unable to conduct this test at the time of the first trial due to stroke-induced expressive dysphasia, severely affecting their ability to produce speech. This subject was included in the study following demonstration of cognitive requirements and consultation with the patient’s stroke physician.

**Figure 1:**
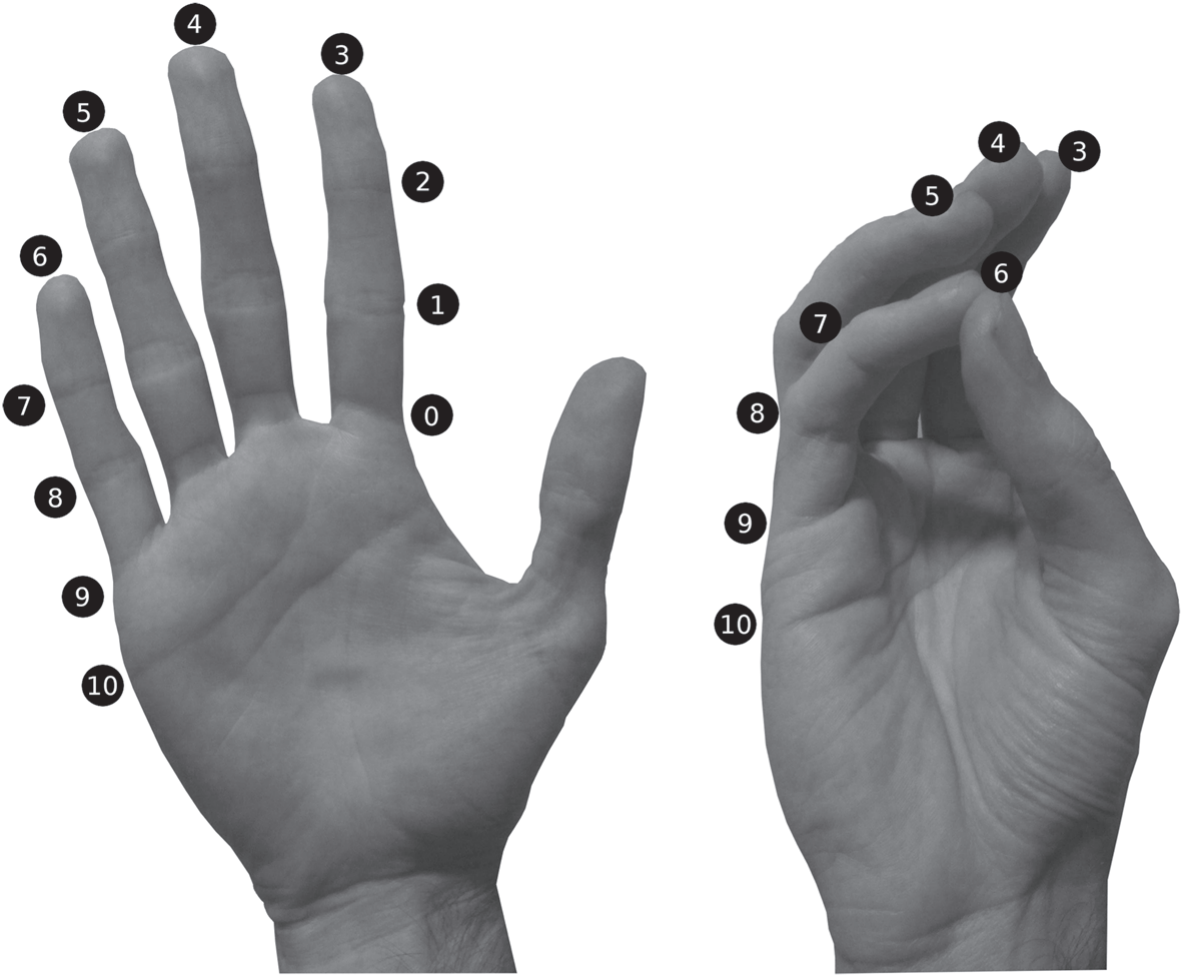
**Kapandji thumb opposition scores.** A score of 0 indicates no opposition, a score of 10 indicates maximal opposition.

The Kapandji finger opposition test [19] was used to determine motor ability in the stroke-affected hand. This test involves the subject attempting to touch the thumb on their stroke-affected hand to 10 points on the same hand in order from points 0 to 10, as shown in Figure 1. Four of the stroke subjects scored at least 6/10, meaning they were able to perform finger tapping with all of their digits. One subject had minimal motor ability in their stroke-affected hand and scored 0/10. Demographic information of the stroke patients, including the MMSE and Kapandji scores at the times of both trials is shown in Table 1. Demographic information of the control subjects is shown in Table 2.

**Table 1:** Stroke subject demographics

				**Early session**					**Late session**	
**ID**	**Sex**	**Dominant**	**Tested**	**Age**	**Time from**	**Kapandji**	**MMSE**	**Time from**	**Kapandji**	**MMSE**
		**hand**	**hand**		**stroke**	**score**	**score**	**early**	**score**	**score**
S1	M	Right	Right	58.8	6w 0d	6/10	28/30	25w 6d	6/10	28/30
S2	M	Right	Right	56.3	3w 2d	6/10	28/30	25w 2d	10/10	28/30
S3	M	Right	Left	75.0	2w 5d	0/10	29/30	25w 1d	3/10	30/30
S4	F	Right	Right	51.9	0w 6d	6/10	27/30	26w 0d	8/10	27/30
S5	F	Right	Right	53.0	3w 0d	9/10	N/A	33w 6d	9/10	28/30

**Table 2:** Healthy subject demographics

**ID**	**Sex**	**Dominant hand**	**Age**
H1	F	Right	75.8
H2	M	Left	43.5
H3	M	Right	61.2
H4	F	Left	67.4
H5	M	Right	40.7
H6	M	Right	71.0
H7	M	Right	50.7
H8	M	Right	21.2
H9	M	Right	71.9
H10	M	Right	68.6

The locations of brain tissue damage due to stroke were varied, including both cortical and subcortical bilateral tissues: the left and right posterior parietal cortex, left frontoparietal cortex, right temproparietal areas, right medial temporal lobe, left thalami and internal capsules, periventricular white matter lesions and centrum semiovale lesions. In all cases, the stroke was ischemic in nature. Subject-specific lesion information can be found in Table 3.

**Table 3:** Stroke subject clinical information

**Stroke subject**	**Lesion information**
S1	Left frontoparietal cortex acute ischemia (left middle carotid artery territory).
S2	Left parietal infarction, left thalami and internal capsule infarcts. Periventricular deep white matter change. Bilateral lacunar infarcts in the centrum semiovale and basal ganglia. 1.5 cm acute infarct in left centrum semiovale.
S3	Area of acute infarction adjacent to the body of the right lateral ventricle involving the right centre of semiovale.
S4	Right posterior parietal and temproparietal regions. Background periventricular ischemic changes involving left frontal parietal region.
S5	Medial right temporal lobe focal infarct. Periventricular deep white matter ischemic disease.

In accordance with ethical requirements, participants were provided with a verbal as well as a written description of this research. Subjects provided written consent to the conduction of the experiment and the publication of their details. In the cases of two stroke patients who were unable to give written consent due to their stroke, verbal consent was accepted. Ethical approval for the experiments was granted by the SJH/AMNCH Research Ethics Committee of the Adelaide & Meath Hospital, Dublin and by the Ethics Committee of the National University of Ireland Maynooth. The experiments were conducted at the Adelaide & Meath Hospital, Dublin.

### Experimental setup and motor paradigm

During a recording session, the subject was seated in a comfortable chair in front of a laptop computer for instruction presentation. The subject was asked to follow on-screen instructions to perform finger-tapping while the words “Move your fingers” were displayed and to rest their hand while the word “Relax” was displayed. Before the first instruction, the screen read “The experiment will begin shortly” while after the final instruction, the screen read “Experiment now over. Please stay still”. The healthy subjects were instructed to perform the task with their dominant hand, while the stroke subjects were instructed to use their stroke-affected hand.

Stroke subjects took part in two recording sessions - an “early” session which took place up to 6 weeks following stroke onset and a “late” session which took place roughly 6 months after the early session. This period of time between early and late sessions was chosen such that spontaneous recovery processes would have had time to run their course. Healthy subjects only participated in one recording session.

The finger-tapping task involved repeatedly touching the thumb to the tips of their 2nd to 5th digits on the same hand at a self-paced speed. During his early recording session, subject S3 was unable to touch his thumb to any other digit yet still had some movement. In this case, the subject still attempted to overtly perform the task.

**Figure 2:**
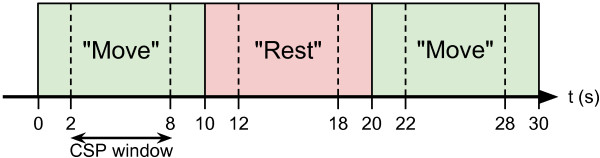
**Experimental protocol.** Experimental protocol diagram showing timings of each trial along with the timing of the window of data used in CSP analysis.

A session consisted of 20 activation trials and 20 rest trials, beginning with activation and alternating until all 40 trials had been completed. Each trial lasted 10 seconds with no rest time between trials (see Figure 2). Subject S3 reported being fatigued during his first recording session and completed only 32 trials.

### EEG data acquisition

EEG data was acquired using a BioSemi ActiveTwo system (BioSemi B.V., Amsterdam, Netherlands) providing 32 Ag/AgCl electrodes positioned according to the 10/20 system. The system also recorded analogue event signals received from the presentation laptop. All data was acquired at a sample rate of 1024 samples per second.

### EEG data analysis

Recorded EEG data was processed off-line in Matlab 7 (Mathworks, Natick, Maine, USA) using a combination of scripts from EEGLAB [20], Ramussen and Williams’ GPML code [21] and custom code.

**Figure 3:**
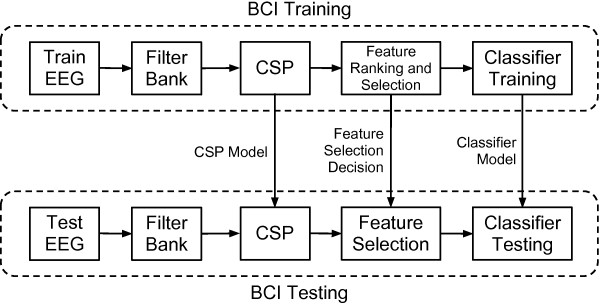
**FBCSP BCI system diagram.** Simplified diagram of the off-line Brain-Computer Interface implementation used.

We implemented an off-line BCI based on Filter Bank Common Spatial Patterns (FBCSP) [22] as illustrated in Figure 3. FBCSP is an adaptation of the Common Spatial Patterns (CSP) algorithm [23]. The general steps of FBCSP are: 

1. Filter the EEG into a number of frequency ranges.

2. Apply the CSP algorithm separately to each frequency range, decompose the EEG and perform feature extraction.

3. Rank and/or select features.

4. Train or test the classifier.

In our case we used Marginal Relevance (MRelv) to rank our features and Gaussian Process Classification (GPC) to classify the selected features. More detail on CSP, MRelv and GPC is provided in following sections.

Two types of BCI training were explored in our investigation: individual and grouped. For individual BCI training, only one EEG dataset was used to train the BCI (“Train EEG”). Training the BCI in this way involved obtaining the CSP filters, ranking and selecting the CSP features and using them to train the classifier, all from a single EEG dataset. Another EEG dataset was used to test the BCI (“Test EEG”). This involved filtering the EEG, applying the trained CSP model, selecting the same features as before and classifying the features with the classifier trained beforehand. For grouped BCI training, all event data from a subset of datasets was used to obtain a general CSP model. As before, this model is applied to all of the training data, the resulting CSP filters are ranked, selected and finally used to train the classifier. This general CSP and feature selection model is then applied to other individual EEG datasets and the resulting CSP features are classified.

Datasets are identified primarily by Subject ID (Table 1 and 2). Stroke subjects took part in an “early” (E) and a “late” (L) session and the datasets from these sessions are labelled accordingly. Therefore, healthy subject datasets are labelled H1–H10, early stroke datasets are labelled S1E–S5E and late stroke datasets are labelled S1L–S5L. Raw data was both visually inspected and analysed for abnormally high signal power to check for any movement artefact that may have affected the trial data. None was found and so no trials were rejected.

#### Pre-processing

In the case of subjects who performed the finger tapping task with their left hand, their EEG data was initially mirrored in the sagittal plane in order to more accurately compare their dominant hand EEG patterns with subjects who performed the task with their right hand.

Raw EEG data was temporally filtered with a filter bank made up of 9 frequency ranges. A zero-phase 4th-order Butterworth filter was used to filter the EEG signals into the frequency ranges 4-8, 8-12, 12-16, 16-20, 20-24, 24-28, 28-32, 32-36 and 36-40 Hz. This filtered EEG was then separated into windowed time segments for each trial of rest and activity. Segments began 2 seconds following the trial onset and lasted 6 seconds as shown in Figure 2.

#### Common spatial patterns

The CSP algorithm [23,24] was then applied to the segments of EEG data from each frequency range. The CSP algorithm produces a set of spatial filters which when used to decompose the EEG signals generates signals whose variances can be used to discriminate optimally between two classes of activity:

(1) $$Z_{b,i}=W_{b}^{T}E_{b,i}$$

where $E_{b,i}\in {\mathbb{R}}^{c_{t}\times t}$ is the *i*th trial EEG measurement from the *b*th frequency range, $W_{b}\in {\mathbb{R}}^{c_{t}\times c_{t}}$ is the CSP projection matrix for the *b*th frequency range, $Z_{b,i}\in {\mathbb{R}}^{c_{t}\times t}$ is the *i*th trial spatially filtered EEG signals for the *b*th frequency range, *c*_*t*_ is the total number of channels, *t* is the number of time samples per trial and *T* denotes the transpose operator.

The feature of an individual trial of data is the logarithm of the proportional variance of one trial compared to all other trials, within each frequency range. Feature extraction and forming of the feature matrix proceeds as:

(2) $$v_{b,i}=\text{log}\left(\frac{\text{var}(Z_{b,i})}{\overset{i_{t}}{\underset{i=1}{\sum }}\text{var}(Z_{b,i})}\right)$$

(3) $${\bar{v}}_{i}=\left[\begin{array}{llll} v_{1,i}^{T}, & v_{2,i}^{T}, & \cdots \;, & v_{b_{t},i}^{T} \end{array}\right]$$

(4) $$\bar{V}=\left[\begin{array}{l} {\bar{v}}_{1}\\ {\bar{v}}_{2}\\ \vdots \\ {\bar{v}}_{i_{t}} \end{array}\right]$$

(5) $$\bar{y}=\left[\begin{array}{l} {\bar{y}}_{1}\\ {\bar{y}}_{2}\\ \vdots \\ {\bar{y}}_{i_{t}} \end{array}\right]$$

where $v_{b,i}\in {\mathbb{R}}^{c_{t}\times 1}$ is the set of features for each trial *i* and frequency range *b*, ${\bar{v}}_{i}\in {\mathbb{R}}^{1\times (b_{t}\cdot c_{t})}$ is the features for each frequency ranges ordered into a single feature vector for each trial, $\bar{V}\in {\mathbb{R}}^{i_{t}\times (b_{t}\cdot c_{t})}$ is the full feature set for all trials, $\bar{y}\in {\mathbb{R}}^{i_{t}\times 1}$ is the true class label vector, *i*_*t*_ is the total number of trials, *b*_*t*_ is the total number of frequency ranges and *c*_*t*_ is the total number of channels.

#### Marginal relevance

Only a selection of CSP features are used for classification training and testing. During our BCI training, CSP features to be used as features for classifier training are ranked and a number of the highest ranked CSP features are selected.

For this study, we chose to use Marginal Relevance (MRelv) as our feature ranking method. The MRelv score for each feature in a feature set ($v_{1}^{T},v_{2}^{T},\cdots v_{b_{t}}^{T}$) is the ratio of their between-group to within-group sum of squares. This idea underpins statistical methodologies such as ANOVA and is explained in more detail elsewhere [25] where it was used to screen out features when a large number of spurious features are present.

The spatial filters for each CSP channel (rows of *W*) have a corresponding spatial filter within the same filter set at a mirrored location within the filter set *W*. Using both filters together offers the best classification results - for example, the 1st and last rows of *W* should be used together or the 3rd and 3rd-from-last rows. Accordingly, we selected the four highest ranked features [22] and their corresponding features for classifier training. This feature selection was then used during the subsequent BCI testing stage.

#### Gaussian process classification

The Gaussian process (GP) model is an example of the use of a flexible, probabilistic, non-parametric model with uncertainty predictions. It fits naturally in the Bayesian modelling framework in which, instead of parameterising a mapping function *f*(*x*), a prior is placed directly on the space of possible functions *f*(*x*) which could represent the nonlinear mapping from input vector *x* to output *y*. Its use and properties for modelling are reviewed in [26,27]. Various applications (e.g. [28,29] in medicine and bioengineering fields) have exploited different properties of GP models for regression problems. In the field of geostatistics GP regression models are used for probabilistic analysis of data and are more commonly known under the term “Kriging”. A GP is a generalization of the Gaussian probability distribution.

Beside regression, GP models can also be used for probabilistic classification [27,30,31]. In the case of classification the output data, *y*, are no longer connected simply to the underlying function, *f*, as in the case of regression, but are discrete. Since the classification is binary, variable *y* can have one value for one class and another for the other class, e.g. *y*∈{1,−1}. The classification of a new data point *x*∗ involves two steps instead of one. In the first step, a latent function *f*, which models qualitatively with a GP model how the likelihood of one class versus the other changes over the x axis, is evaluated. In the second step, the output of the latent function *f* is squashed onto the range [0, 1] using any sigmoidal function, *π*(*f*)=*prob*(*y*=1|*f*). This means that the squashed output of GP model represents the probability of a data point belonging to one of two types.

The result then, after classification, is that each event is assigned a probability value in the range [0, 1] where a score of 0 indicates complete confidence that the event belongs to one class and a score of 1 indicates complete confidence that an event is of the other class. In practice, the majority of events take intermediate values. We applied a decision threshold of 0.5 to the probability scores to determine which class an event had been classified as belonging to by GPC.

### Analyses

The first analysis carried out was 10-fold cross-validation on each dataset. Trials were split into 10 subgroups, separated in temporal order. Nine of the subgroups were used for: *(1)* training the FBCSP model, *(2)* selection of the top ranking features using MRelv and *(3)* training the GPC model. For the remaining subgroup, the previously created FBCSP model was applied with the same features selected as determined by MRelv and those features were then classified by the GPC model. This was the repeated for each of the 10 subgroups in a dataset. The purpose of this analysis is to establish the consistency of the EEG responses and the classification features derived during processing. A poor average classification result would indicate that the responses recorded in a dataset were inconsistent and thus possibly unsuitable for deriving a general response.

Individual BCI training was carried out using individual healthy datasets. Each dataset, including the healthy ones, were then tested using each of the trained BCIs. This resulted in a set of one-on-one BCIs, where one subject’s EEG patterns were classified against each of the healthy subject’s EEG patterns. Although this is an atypical BCI modality approach, it allows us to see how the classification rates vary between subjects.

Grouped BCI training was also carried out using all of the healthy datasets. A general BCI was trained from the 10 healthy subjects. Each stroke subject dataset was individually tested against this general BCI. This is a common implementation for communications BCI and so is useful for our investigation. Furthermore, these classification results are useful for comparison to the individual BCI results obtained earlier. Similarly, we carried out Leave-One-Out Cross-Validation on the healthy datasets. All but one healthy dataset were grouped to train a BCI and the excluded healthy datset was tested against this model. This was then repeated for each healthy dataset. These classification results are useful for comparison with the stroke-affected results.

Individual BCI training was performed for each subject where the early dataset was used to train the BCI and then the same subject’s late dataset was then tested on that BCI. The comparison of the results from this analysis with the results from training the BCI on healthy EEG patterns are important to our investigation.

Another result of interest is the frequency ranges of selected CSP features for each dataset. To investigate this, the frequency ranges of the selected CSP features was recorded. For each group of Healthy, Early Stroke and Late Stroke, we obtained a histogram of selected frequency ranges to see which were favoured and highlight any differences between groups.

## Results

### Single dataset 10-fold cross validation

The classification results following 10-fold cross-validation on each dataset are shown in Table 4. 8/10 healthy subject datasets scored 100% and the remaining 2/10 scored 97.5% while only 5/10 stroke datasets scored 100% and the remaining 5/10 scored between 85% and 97.5%. There is no distinction between the early/late stroke datasets as 2/5 early stroke datasets scored 100% while 3/5 late stroke datasets scored 100%. We tested a range of k values for k-fold cross validation of k = 2, 4, 6... 16. We saw no significant changes in these results compared to k = 10.

**Table 4:** Single dataset 10-fold cross-validation classification accuracy%

Dataset	S1E	S1L	S2E	S2L	S3E	S3L	S4E	S4L	S5E	S5L
Accuracy	97.50	100.00	100.00	87.50	93.75	100.00	100.00	85.00	95.00	100.00
Dataset	H1	H2	H3	H4	H5	H6	H7	H8	H9	H10
Accuracy	100.00	100.00	97.50	97.50	100.00	100.00	100.00	100.00	100.00	100.00

### Individual healthy dataset models applied to all data

A table of individual classification accuracies when training the models and classifier on each healthy dataset and then testing on all other datasets is presented in Table 5.

**Table 5:** Cross-dataset classification accuracy%

**Test EEG dataset**	**Train EEG dataset**												
		**H1**	**H2**	**H3**	**H4**	**H5**	**H6**	**H7**	**H8**	**H9**	**H10**	**Avg.**	**StDev**
S1E		62.5	75.0	97.5	85.0	72.5	47.5	87.5	90.0	87.5	72.5	77.8	14.9
S1L		75.0	70.0	80.0	75.0	42.5	77.5	57.5	70.0	82.5	67.5	69.8	11.9
S2E		70.0	62.5	60.0	77.5	50.0	62.5	70.0	50.0	87.5	65.0	65.5	11.5
S2L		57.5	50.0	55.0	55.0	40.0	55.0	60.0	52.5	62.5	57.5	54.5	6.2
S3E		75.0	53.1	81.3	75.0	62.5	78.1	84.4	78.1	81.3	75.0	74.4	9.5
S3L		77.5	60.0	87.5	72.5	52.5	85.0	70.0	80.0	92.5	90.0	76.8	13.1
S4E		70.0	57.5	40.0	65.0	60.0	65.0	67.5	52.5	62.5	72.5	61.3	9.5
S4L		52.5	70.0	40.0	50.0	55.0	70.0	47.5	55.0	60.0	57.5	55.8	9.4
S5E		90.0	55.0	65.0	90.0	75.0	80.0	55.0	72.5	65.0	72.5	72.0	12.5
S5L		82.5	57.5	60.0	77.5	80.0	55.0	47.5	85.0	70.0	95.0	71.0	15.4
H1			72.5	75.0	92.5	97.5	97.5	87.5	100.0	97.5	100.0	91.1	10.6
H2		62.5		37.5	62.5	80.0	55.0	67.5	90.0	55.0	82.5	65.8	16.3
H3		95.0	65.0		85.0	80.0	65.0	87.5	95.0	80.0	90.0	82.5	11.3
H4		80.0	72.5	77.5		57.5	65.0	77.5	82.5	90.0	90.0	76.9	10.7
H5		85.0	72.5	55.0	52.5		52.5	65.0	80.0	60.0	85.0	67.5	13.5
H6		100.0	65.0	65.0	97.5	87.5		80.0	70.0	95.0	87.5	83.1	13.7
H7		92.5	65.0	92.5	82.5	87.5	72.5		92.5	92.5	65.0	82.5	11.9
H8		100.0	80.0	60.0	90.0	90.0	80.0	72.5		90.0	92.5	83.9	12.1
H9		95.0	55.0	67.5	97.5	80.0	92.5	65.0	100.0		97.5	83.3	17.0
H10		87.5	67.5	80.0	85.0	85.0	87.5	70.0	95.0	100.0		84.2	10.5
Healthy datasets												80.1	14.4
All stroke datasets												67.9	13.7
Early stroke datasets												70.2	12.8
Late stroke datasets												65.6	14.3

Wilcoxon Rank Sum tests were used to evaluate statistical differences between these classification results for the Healthy, All Stroke, Early Stroke and Late Stroke groups. There were significant differences found between Healthy (Median = 82.5) and All Stroke (Median = 70.0) (*Z*=5.55,*p*<0.05,*r*=0.40), between Healthy (Median = 82.5) and Early Stroke (Median = 71.25) (*Z*=−3.97,*p*<0.05,*r*=0.34) and between Healthy (Median = 82.5) and Late Stroke (Median = 61.25) (*Z*=−5.18,*p*<0.05,*r*=0.44). There was no significant difference found between Early Stroke (Median = 71.25) and Late Stroke (Median = 61.25) (*Z*=1.75,*p*>0.05,*r*=0.17).

### Grouped healthy dataset model applied to stroke data

Classification accuracies of each stroke dataset when the BCI is trained on all of the healthy EEG datasets grouped together is presented in Table 6.

**Table 6:** Grouped healthy classification accuracy%

**Test dataset**	**Accuracy**
S1E	87.5
S1L	62.5
S2E	52.5
S2L	55.0
S3E	75.0
S3L	87.5
S4E	65.0
S4L	65.0
S5E	82.5
S5L	90.0
All average	72.3±14.1
Early average	72.5±14.0
Late average	72.0±15.8

Wilcoxon Signed Rank tests were used to test for statistical significance in the change in classification accuracy when using grouped healthy datasets to train the BCI as compared to the average results when using individual healthy datasets to train BCIs. We found no significant change (*p*>0.05) in classification accuracy for datasets S1E, S1L, S2L, S3E and S4E. We found significant increases (*p*<0.05) in classification accuracy for datasets S3L, S4L, S5E and S5L and a significant decrease (*p*<0.05) for dataset S2E.

### Leave-one-out cross-validation of healthy datasets

Classification accuracies of each healthy dataset when the BCI is trained on all other healthy datasets grouped together is presented in Table 7.

**Table 7:** Leave-one-out cross-validation of healthy data classification accuracy%

**Test dataset**	**Accuracy**
H1	100.0
H2	90.0
H3	95.0
H4	80.0
H5	87.5
H6	97.5
H7	92.5
H8	95.0
H9	95.0
H10	85.0
Average	91.8±6.1

Wilcoxon Rank Sum tests were used to evaluate statistical differences between these classification results for the Healthy, All Stroke, Early Stroke and Late Stroke groups when grouped healthy datasets were use to train the BCI. There was a significant difference found between Healthy (Median = 94) and All Stroke (Median = 70) (*Z*=3.04,*p*<0.05,*r*=0.68), between Healthy (Median = 94) and Early Stroke (Median = 75) (*p*<0.05) and between Healthy results (Median = 94) and Late Stroke results (Median = 65) (*p*<0.05). There was no significant difference found between Early Stroke results (Median = 75) and Late Stroke results (Median = 65) (*p*<0.05). The *Z* and *r* statistics were not calculated when very few data points were available. These between-group significant difference results are the same as those obtained when using individual BCIs trained on healthy datasets.

### Early Stroke datasets used to classify corresponding Late Stroke datasets

Classification results of Late Stroke datasets when training with the corresponding Early Stroke dataset are shown in Table 8. Classification accuracy of the five Late Stroke datasets ranged from 62.5% to 95% with a median of 75.0%. We can compare these classification accuracy results to those obtained when training the BCI on individual healthy datasets and those obtained when training on grouped healthy datasets.

**Table 8:** Longitudinal classification accuracy%

**Training dataset**	**Test dataset**	**Accuracy**
S1E	S1L	82.5
S2E	S2L	72.5
S3E	S3L	95.0
S4E	S4L	62.5
S5E	S5L	75.0
	Average	77.5±12.1

Wilcoxon Signed Rank tests were used to compare these longitudinal classification results to those obtained when using BCIs trained on individual Healthy datasets. A significant (*p*<0.05) increase was seen for S1L, S2L and S3L. There was no significant change (*p*<0.05) found for S4L and S5L.

Comparing the longitudinal classification accuracies to those obtained when training the BCI on grouped healthy datasets, we see that S1L improved from 62.5% to 82.5%, S2L improved from 55% to 72.5%, S3L improved from 87.5% to 95%, S4L reduced from 65% to 62.5% and S5L reduced from 90% to 75%.

A table of collated classification results of each BCI training method for each stroke dataset is presented in Table 9.

**Table 9:** Comparison of BCI training methods for stroke classification

**Dataset**	**Individual healthy**		**Grouped healthy**	**Early stroke**
	**Avg**	**StDev**		
S1E	77.8	14.9	87.5	
S1L	69.8	11.9	62.5	82.5
S2E	65.5	11.5	52.5	
S2L	54.5	6.2	55.0	72.5
S3E	74.4	9.5	75.0	
S3L	76.8	13.1	87.5	95.0
S4E	61.3	9.5	65.0	
S4L	55.8	9.4	65.0	62.5
S5E	72.0	12.5	82.5	
S5L	71.0	15.4	90.0	75.0
All average	67.9±8.3		72.3±14.1	
Early average	70.2±6.7		72.5±14.0	
Late average	65.6±9.9		72.0±15.8	77.5±12.1

### Frequency ranges of selected CSP features

**Figure 4:**
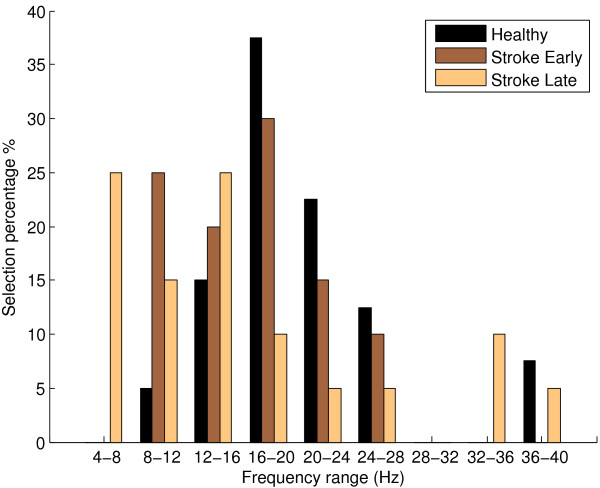
**Selected CSP feature frequency ranges.** Histogram of frequency ranges of selected CSP features following Marginal Relevance ranking for each for the groups Healthy, Stroke Early and Stroke Late.

Presented in Table 10 are the frequency ranges of the CSP features selected for classifier training for each full dataset. We also present a corresponding histogram of this data grouped for Healthy, Stroke Early and Stroke Late datasets in Figure 4. This histogram suggests that, for healthy EEG, the frequency ranges of the CSP features in the 16-20 Hz and 20-24 Hz are most frequently selected. Early stroke datasets display some of the healthy datasets’ preference for selection of features in the 16-24 Hz range however there is also increased selection of features in the 8-16 Hz range. Late stroke datasets appear to shift towards further selection of CSP features in lower frequency ranges, with a noticeable increase in selection in the 4-16 Hz range and a relative decrease in selection from 16 Hz upwards.

**Table 10:** Frequency ranges (Hz) of selected CSP features for each dataset

**Dataset**			**Rank of selected features**	
	**1st**	**2nd**	**3rd**	**4th**
S1E	16-20	12-16	8-12	16-20
S1L	8-12	4-8	8-12	4-8
S2E	8-12	8-12	12-16	20-24
S2L	36-40	32-36	32-36	12-16
S3E	12-16	8-12	16-20	20-24
S3L	12-16	12-16	16-20	12-16
S4E	24-28	8-12	16-20	20-24
S4L	24-28	12-16	20-24	16-20
S5E	16-20	12-16	24-28	16-20
S5L	4-8	4-8	8-12	4-8
H1	16-20	20-24	16-20	16-20
H2	24-28	36-40	20-24	24-28
H3	36-40	16-20	24-28	36-40
H4	16-20	16-20	20-24	16-20
H5	20-24	24-28	20-24	16-20
H6	12-16	8-12	12-16	12-16
H7	12-16	16-20	16-20	12-16
H8	20-24	16-20	20-24	24-28
H9	12-16	16-20	8-12	16-20
H10	20-24	16-20	20-24	16-20

## Discussion

Our first analysis result following 10-fold cross-validation demonstrated that stroke-affected EEG is more likely to contain individual trials misclassified than healthy EEG. We can speculate on possible reasons why this is so.

For example, it is possible that EEG patterns from a stroke-affected brain are more variable and are less stable than those from a healthy brain, even if the stroke subject is consistent in their motor task. Given that even with healthy subjects engaging successfully in a motor task, flawless classification is not always possible then it is not unreasonable to expect similar or even worse consistency in stroke-affected brains. It is also possible that these mis-classifications are due to the subject mis-performing the task. A lapse in concentration on the part of the subject, a restless hand movement, an involuntary leg twitch or possibly the effects of fatigue could reasonably cause a change in the event-related EEG confounding the efforts of the classifiers.

We make the assumption that each subject performed the task correctly and to the best of their ability. Visual supervision of the subjects did not reveal any movement incidents and neither did our artefact analysis of trial data. One aspect of recording experimental data with stroke subjects is that minimization of preparation time and set up is important to reduce the likelihood of a subject becoming fatigued and being unable to complete the task. Therefore screening for extraneous muscular artefact based on recording activity of other peripheral muscle groups with, for example, electromyography (EMG) would add substantially to the instrumentation set up burden as well as risk the further discomfort of the stroke patient. Incidentally, subject S3 reported being fatigued during their early experimental session, resulting in only 36 out of the potential 40 trials being completed. Dataset S3E also scored the 2nd lowest 10-fold cross-validation classification rate of all datasets at 93.75%. This may suggest a link between fatigue and low k-fold cross-correlation result but that the lowest scoring dataset was S4L, where no fatigue was reported. This illustrates the difficulty of describing the processes which underlie the variable EEG features identified.

Three options for training a BCI were analysed. The first two, training on healthy EEG and testing on stroke EEG, represent zero-training BCI methods - an important consideration for stroke rehabilitation BCI. In one case, we trained a BCI for each healthy dataset and in the second, we trained a single BCI on all healthy datasets grouped together. This latter method is similar to a general BCI used for communication and control. The former method, however, provides more information relating to individual training and testing datasets. We can see, for example, in Table 5 that dataset S1E was classified quite well with the healthy dataset H3 (97.5%) yet was classified poorly with the healthy dataset H6 (47.5%). These cross-dataset EEG classifications are important because the reasons for such varying classification successes may be important for advancing rehabilitation BCI and our understanding of stroke-affected EEG, yet these are not results that we would see if we restricted ourselves to the more typical general BCI method. A full investigation into these potential reasons is beyond the scope of this study yet may be very interesting and useful future work.

Although training numerous BCIs on each healthy EEG dataset is useful for exploring aspects of stroke-affect EEG for BCI, they do not represent a real-world implementation of a zero-training BCI. This is the purpose of the single general BCI trained on grouped healthy EEG datasets. With this, we can see how well individual stroke EEG patterns would be classified in a zero-training scenario. We find that classification rates differ significantly from the average classification rates of the individual BCIs in 5/10 stroke datasets (S2E, S3L, S4L, S5E and S5L), with 4/5 displaying an improvement. We can see how classification rates of subject’s EEG patterns change from the early session to the late session. Some subjects see a marked increase (S3 and S5), some see little change (S2 and S4) and one sees a marked decrease (S1). These results suggest that the EEG signal space related to the motor task alters significantly over time, in at least some stroke cases. The results of accuracy measurements reported here may be useful in characterizing the change in EEG activity patterns during the recovery phase following stroke and so may potentially be used as a measurement of the magnitude of neuroplasticity and compensatory changes in the brain’s motor networks.

We suspect that these changes are due to numerous unmeasured factors, such as lesion location, patient physical rehabilitation or the patient’s typical use of the stroke-affected hand. Although we have a measure of each subject’s Kapandji score relating to their hand movement capabilities, we have not attempted to relate this to a subject’s classification accuracies or their change in classification accuracies over time. While Kapandji score, or other measures of stroke-affected movement, may be related, we do not have a large enough dataset to attempt to make a connection.

For the late stroke datasets, we can compare classification results for the third scenario where the BCI has been trained on that subject’s own EEG recorded 6 months previously. Table 9 presents the classification results of all three methods and shows us that for 4/5 late stroke datasets, training the BCI on the early stroke dataset provides the best classification accuracy. There are some interesting points of discussion here regarding whether to train a rehabilitation BCI on healthy EEG patterns or a subject’s own previously recorded EEG patterns. Firstly, using the healthy EEG datasets, classification results are lower. This may lead to frustration for the stroke patient, resulting in non-compliance with rehabilitation BCI therapy, even though the EEG patterns the patient must generate reflect those typical of healthy cortex. Secondly, training on the early stroke EEG patterns could potentially result in a less frustrating experience and better engagement from the patient, improving their rehabilitation outcomes. Unfortunately, as we have seen in Figure 4, the early stroke EEG patterns are not characteristic of EEG from a healthy brain reflecting, most likely, the network pathophysiology resulting from the stroke. It seems that the advantages and disadvantages of training a rehabilitative BCI on either general healthy EEG or a subject’s own earlier recorded EEG will have to be considered. We feel that this will be an important question to be answered for this field of research.

**Figure 5:**
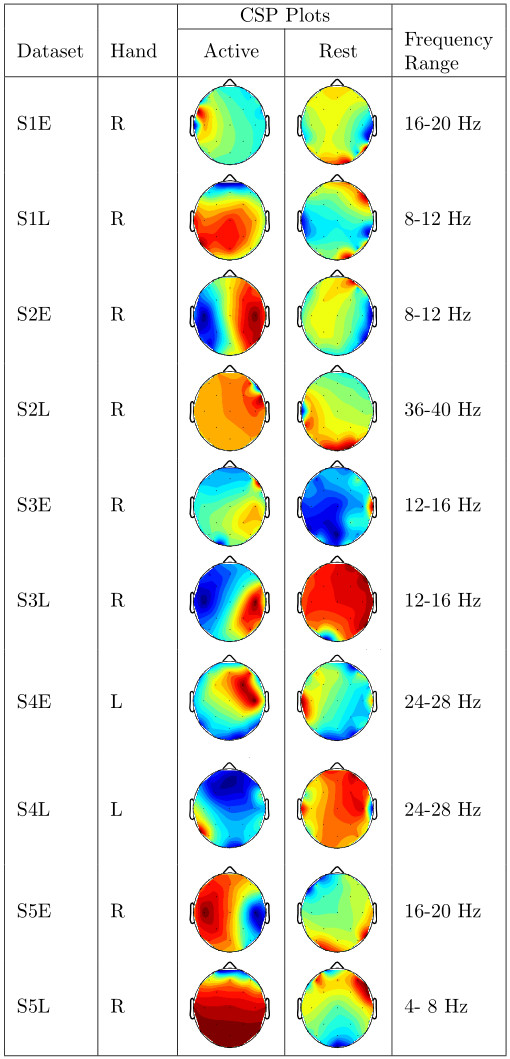
**Stroke CSP plots.** Plots of the highest-ranking common spatial patterns (columns of *W*^−1^) for each stroke dataset along with the frequency range the CSP plot belongs to.

**Figure 6:**
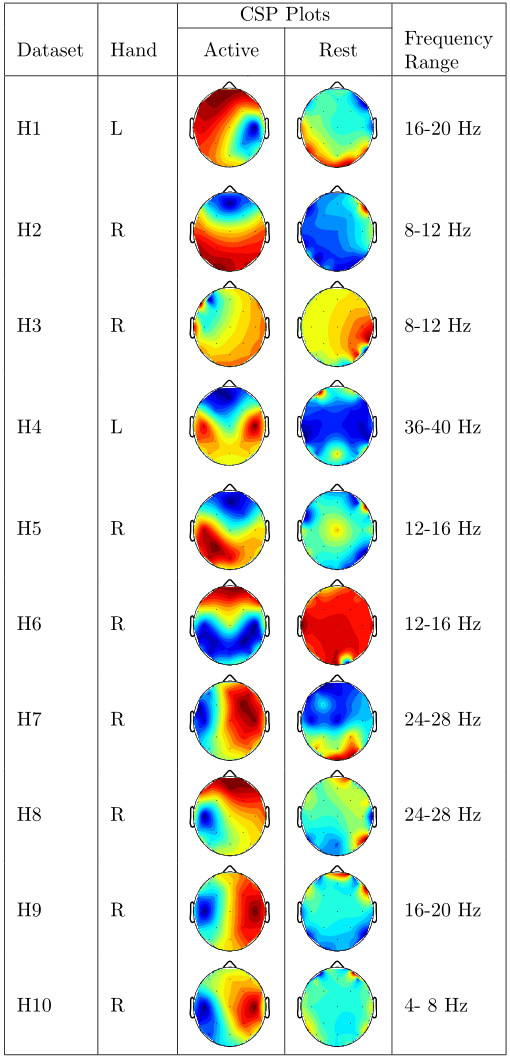
**Healthy CSP plots.** Plots of the highest-ranking common spatial patterns (columns of *W*^−1^) for each healthy dataset along with the frequency range the CSP plot belongs to.

Presented in Figure 5 and Figure 6 are CSP plots of the highest ranked CSP features for both classes of activity for all datasets. Unfortunately we have far too few datasets for these plots to provide more than a qualitative analysis of the differences between stroke-affected and healthy CSP plots. It appears that there is more left/right asymmetry in the common spatial patterns of healthy datasets than stroke-affected datasets. As the differences between the two groups is not strong enough to draw any conclusions, we instead feel that these plots suggest that stroke-affected CSP plots are not dissimilar to healthy CSP plots. Perhaps with a much larger dataset a thorough analysis of the differences between stroke-affected and healthy CSP plots would be possible.

The decision to record a session of EEG activity to train a BCI for each subject may also depend on a trade-off between improved classification accuracy and any possible negative effects of subjecting a stroke patient to an EEG recording session. Possible negative effects include anxiety (as many stroke patients are elderly and may have apprehension about participating in an EEG recording session), loss of therapy time (as time spent training leads to a reduction in time spent using the BCI in a therapeutic mode) and fatigue (because a stroke patient may become fatigued as a result of training, leaving little energy for the therapeutic interaction). In these patients where the above factors are prevalent the BCI may have to be trained using healthy data. The disadvantage of this approach from a therapy perspective is that the inferior performance of the classifier may lead to frustration on the part of the patient and a potential rejection of the therapy.

Given the changes in the EEG pattern in stroke compared to the stereotypical patterns for healthy subjects and their evolution over time it is clear that there is considerable scope for improved machine learning techniques which can work from short session data and continually adapt to the user. There is some recent work in this area for healthy subjects using passive movement approaches [32] and data space adaptation techniques [33]. However, we wish to remark here that it is incredibly important to note the tension between using machine learning to adapt the interface to the EEG patterns on one hand and forcing the patient to adapt to a classifier which is targeting the appropriate cortical networks for healthy movement on the other. To understand this somewhat subtle point, it is worth noting that natural recovery in stroke is often suboptimal (spasticity, abnormal muscle synergies, etc.) and these neurological symptoms can be related to pathophysiological motor and compensatory networks that have arisen from the reorganization process. It is these changes which are most likely reflected in the EEG measurements reported here. If a machine learning algorithm consistently adapts to the patient to optimize communication with the feedback interface the therapy may well lead to reinforcement of these maladaptive changes. It may be better that the patient adapts to a classifier which is set up to expect EEG features which are more typically associated with engagement of those areas of cortex more associated with healthy movement. The catch is that such a classifier may be far too frustrating to use and therefore some trade-off between encouraging engagement and directing recovery will have to be met for an effective BCI instrument in this use case scenario. This issue should be contrasted with the corresponding case for communicative BCI which instead adapts to whatever aspects of a subject’s EEG is under volitional control requiring less adaptation on the part of the user.

In terms of the machine learning options, Gaussian Process classification was our chosen method because, as an alternative to the more commonly used method of Naive Bayesian classification for BCI, GP classification makes no assumptions about the underlying class boundary between regressors, including allowing for non-linear class boundaries. As we are working with stroke-affected EEG, we feel that this is a more robust classification method to use when we are uncertain of the class space. At the other extreme, neural networks would provide the most detailed class boundary. However, GP classification requires optimization of relatively few parameters compared to neural networks. We see this as an advantage over both Naive Bayesian classification and neural networks. We wish to explore this method further as part of our ongoing investigation into its usefulness for BCI applications. Finally, GP classification produces more information than that reported in this study and this could potentially be used for gaining deeper insight into the variability of the features. As stated in the description, GPC does not simply return a binary class membership but a probability of class membership. We applied a decision threshold to this probability but the probabilities themselves are information that could potentially be explored in depth. After initial investigations, we found that there is a notable difference in the variance of class membership probabilities for stroke patients compared to healthy subjects. This is an investigation that we are carrying out presently.

## Conclusions

Rehabilitative BCIs must take into account the difference in EEG patterns between healthy subjects and stroke-affected subjects in order for the system to be effective and to aid in recovery. The ideal scenario of a zero-training rehabilitative BCI is possible using healthy EEG but the classification accuracy is lower than for healthy subjects which could be excessively frustrating for patients. Classification accuracy of stroke EEG is improved significantly through subject–specific BCI training sessions even 6 months prior however this comes with a cost in terms of loss of rehabilitation time and potentially over-adaptation to the user, which may be detrimental in terms of optimal recovery. It is clear that a rehabilitative BCI must have different technical requirements to those for a communication and control BCI and these differences must be considered when developing the appropriate machine learning scheme for this use case.

## Competing interests

The authors declare that they have no competing interests.

## Authors’ contributions

DJL designed and coordinated the study, performed analysis, interpreted the data and drafted the manuscript. TEW conceived of, designed and coordinated the study, interpreted the data and drafted the manuscript. JD collected the neuropsychological and EEG data and contributed text. SC and RAPR collected the neuropsychological and EEG data. JK and KD performed analysis and contributed text. DRC was patient clinical supervisor. All authors read and approved the final manuscript.
